# ADULT CHILDREN’S UNDERSTANDING OF PARENTS’ CARE AND LIVING PREFERENCES AT END OF LIFE

**DOI:** 10.1093/geroni/igz038.2473

**Published:** 2019-11-08

**Authors:** Meghan McDarby, Matthew Picchiello, Elissa K Kozlov, Catherine Ju, Dani Worthalter, Brian Carpenter

**Affiliations:** 1 Washington University in St. Louis, St. Louis, Missouri, United States; 2 Rutgers University, New Brunswick, New Jersey, United States

## Abstract

Adult children who are uninformed about their parents’ preferences for end-of-life care may not be prepared to advocate on their behalf when the circumstances arise. The purpose of the current analysis was to examine how well adult children understand their parents’ end of life preferences. We analyzed responses from adult children (n = 70) of 40 older adults (65+) who participated in an intervention to improve family communication about end-of-life care. We compared children’s and parents’ responses on the same set of 6 questions about healthcare decision-making (e.g., “Which medical decision-maker has the final say?”) and 4 questions about living preferences (e.g., “Would you want to move to a nursing home?”). We also examined demographic differences between children who had higher agreement (≥ 6/10 questions correct; n = 32) versus lower agreement (<6/10 questions correct; n = 38). On average, children provided the same response as their parents on approximately 5 out of 10 questions. Overall, adult children answered more questions correctly about living preferences compared to preferences about healthcare decisions (t(69) = 6.59, p < 0.001). In terms of demographic characteristics, there were no significant differences between children with higher and lower agreement with their parents’ preferences on variables including gender, frequency of contact with parents, and living proximity to parents. Our results underscore the need for increased communication between adult children and their parents about topics likely to influence quality of care at end of life.

